# Specific Factors That Influence Adherence to Beta Blocker Treatment in Primary Prevention of Variceal Bleeding in Cirrhotic Romanian Patients. a Proof of Concept Qualitative Study

**DOI:** 10.25122/jml-2018-1006

**Published:** 2018

**Authors:** Silvia Sovaila, Adrian Purcarea, Dan Gheonea, Tudorel Ciurea

**Affiliations:** 1. Gastroenterology Department, University of Medicine and Pharmacy, Craiova, Romania; 2. Internal Medicine Clinic “Internist.ro”, Brasov, Romania

**Keywords:** adherence, beta blockers, cirrhosis, qualitative

## Abstract

Adherence to medical treatment in chronic patients challenging, especially in primary prevention where the benefit is not directly perceived by the patient. Through the directed interview of this qualitative study we assessed some specific factors that intervene in beta-blockers adherence in cirrhotic patients with oesophageal varices in primary prevention in Romanian. We identified that the most important modifiable extrinsic factor that influences adherence is the confidence in medicine. The intensely negative perception of the disease seemed to be another major driver of adherence.

## Introduction

Chronic diseases imply that patients will take long-term treatments. Adherence has been defined as “the consequence of a process of a patient’s conforming to medical instructions issued by the medical authority in the framework of treating his illness” [1]. Studies show great limits to patients’ adherence [2], and researchers actively try to identify the factors that influence it [3]. Too often, there is a trend to blame patients for not respecting their treatment; however, the determinants of adherence are numerous and interdependent. There is evidence that providers and healthcare systems can have significant influence at this level. The intervention in adherence may have a greater impact on health than any improvement of treatments [4].

Previous research focused more on interventions in how to increase adherence, alas with limited efficacy as shown by the first systematic review by Haynes RB in 1996. At that time, interventions were considered complex and not cost-efficient [5]. In 2008, the latest Cochrane systematic review and meta-analysis on adherence brought no more new information on how to help our patients [6].

Cirrhotic patients who are of particular interest to us suffer from low adherence [7, 8]. Moreover, when the direct benefit is not visible – like in primary prevention of variceal bleeding – the risk of inobservance seems even greater [9].

The present study aims to isolate some of the modifiable subjective and objective factors that might influence adherence to beta-blocker primary prophylaxis in Romanian cirrhotic patients, to better understand how to approach our patients.

## Materials and Methods

This is an observational monocentric study, consisting of a series of semi-structured interviews targeting cirrhotic patients.

The primary objective is to evaluate the impact of disease awareness and confidence in medicine on prophylactic beta-blocker therapy observance in cirrhotic patients with oesophageal varices.

Inclusion criteria: Patients who are over 18, have a definite cirrhosis diagnosis and proven oesophageal varices and undergo prophylactic beta-blocker treatment prior to inclusion. Patients were excluded if past or present hemorrhagic event was identified or they declined consent.

## Data Collection and Analysis

The interviewer was not a treating physician. We used semi-directed recorded interviews. Interviews, defined as a conversation, were face-to-face and in-depth, directed by open questions posed by the researcher in order to elicit the participant’s experiences, perceptions, thoughts, and feelings. The interview questions followed an interview guide and lasted between 10 to 15 minutes. They included 8 open questions, aimed to assess the level of understanding of the impact of treatment on morbidity and mortality induced by the disease, the impact of treatment on quality of life, tolerance to treatment, and the degree of confidence in the treatment and in the modern medicine and the use of alternative medicines (Table 1).

The interviews in an audio format were processed by the first investigator (SS) into text format and the accuracy of the conversion was assessed by the second investigator (AP). Keyword coding with a simple analysis was performed with Weft QDA 2.0 software, according to treatment adherence.

### Treatment observance

Treatment observance was established by direct questions to the patient and was confirmed with the treating physician.

### Informed consent, privacy

The purpose and the methodology of the study were clearly explained, especially the fact the conversations were recorded. Names and other data that would allow identification of the data subjects were not recorded. All patients enrolled in the study expressed their written informed consent.

Statistical Analysis. Inter-observer reliability was computed using Cohens’ Kappa in SPSS 22 (IBM Corp).

## Results

The study was conducted in July 2015, for one week, in County Clinical Emergency Hospital of Craiova, Gastroenterology ward. We screened 10 potential candidates within the hospitalized cirrhotic patient population, who were already on beta blocker therapy prior to admission. One candidate had an exclusion criterion (recent active variceal bleeding) and 3 others declined participation. We conducted 6 semi-directed interviews. The mean age was 67 [50:82] with a gender repartition of 4 men and 2 women.

Out of the 6 patients, 5 reported good adherence to beta blocker therapy, later confirmed by the treating physician.

## Interviews

According to the method of semi-directed interviews, the questions were structured around 5 categories: Disease comprehension, Disease perception, Treatment impact, Reliance on alternative medicine, and Confidence in medicine. Coding is available as supplementary data.

Disease comprehension was found to be minimal to moderate for most patients – 5 out of the 6 – with answers mainly as “it is a liver disease”. Only one patient had a good comprehension with a moderate, realistic perception (“an advanced liver disease that is fibrotic and can develop various conditions and evolves in different stages”).

Disease perception: varied between very negative (rapid global degradation expected –“very bad evolution”), to less negative (“it will not evolve very rapidly if treated”), and lack of interest from probable deflection cooping (“I do not really think about the disease”).

Treatment impact was moderate (“I feel they do me some good”) to minimal (“I feel the same”). No adverse events were noted; thus, the impact on adherence is unknown. Treatment knowledge – function and expected benefit – was overall lacking (“for the liver, I do not know”).

Confidence in medicine was positive in two patients: “I have enough confidence” or “I have confidence, I died and was resurrected” and limited for the rest of patients, ranging from “with our economic power…” to “what else can I do” and “I have no choice”. Reliance on alternative medicine was present in 2 patients of whom one acknowledges substituting medicines with herbal infusions. The other was currently less convinced by alternative medicine after a trial and error process “I saw that I took them for nothing”, “They had no effect”.

Inter-observer variability. Interviews were processed and analyzed separately by two independent observers (SS and AP). The results were coded into categories with a good to excellent inter-observer reliability (supplementary data, in Romanian) (Cohen Kappa = 0.78).

**Table 1:** Type and aim of the interview questions.

Questions	Aims
1. What do you understand by the term ‘cirrhosis’?	Disease comprehension and disease impact
2. What do you understand by the term ‘oesophageal varices’?	Disease comprehension
3. What do you think is the evolution of the disease (cirrhosis)?	Disease perception, comprehension and disease impact
4. What role do beta blockers have (propranolol or carvedilol)? Why do you take them?	Disease comprehension
5. Have you felt any change since you started taking them?	Treatments comprehension and perception
6. Have you noticed any adverse effect? What type?	Treatments effect/side effects
7. How much trust do you have in medicine/doctors?	Medicine trust
8. Have you been appealing to alternative treatments (infusions, natural supplements, ancient remedies)?	Medicine trust
9. Did you take your treatment during the last month (propranolol, carvedilol). Why?	Treatment observance. Disease perception and comprehension. Disease impact

## Discussions

Improved adherence to treatment in chronic patients brought a substantial reduction in healthcare costs [10] with an outcome difference of 26% when comparing high versus low adherence in chronic patients in a meta-analysis [11]. In Romanian settings, specific local factors were not pointed out by previous studies. We aim to identify modifiers of adherence to beta blocker treatment somewhat specific to Romanian cirrhotic patients.

The lack of knowledge was globally present. This is not a local problem, a study on Australian cirrhotic patients found that only half of the patients remembered having information of their disease and a majority reported difficulty in understanding the medical information [12]. There is a lack of research on the effectiveness of therapeutic patient educational interventions for improving patient adherence [13]. Finding ways to make our patients understand their disease and the therapeutic implications is not an easy task, especially when cultural factors, like a patriarchal physician-patient relation, limited interactions [14]. In our study, adherence was only partially influenced by the disease comprehension, while others found that knowledge stood out as a significant factor that influences adherence [15, 16].

We found that the negative perception of the disease was a substantial factor that increases adherence. There is not enough research to elucidate the relationship between illness perceptions and patient medication adherence [17]. In two studies on anti-hypertension medication, adherence was increased in the patients with a low personal sense of control over the disease [18, 19], while the negative perception of the disease and the belief in the personal ability to control illness are probably interrelated.

Our patients did not fully understand the need for treatment. Treatment (of a symptomatic disease) and prophylaxis seem to be perceived as two completely different entities. In primary prophylaxis, where the clinical benefit is not readily visible, the perceived “usefulness of the treatment” was a low adherence factor [20].

Traditionally, confidence in medicine is limited in Romania due to the old medical infrastructure, inequitable access and the negative media coverage [21], but a new, positive global direction is beginning to show. In our study, patients seemed to be neutral towards medicine. They thought not to have other alternatives but to follow medical instructions. However, confidence was particularly low in inobservant patients.

Advertising campaigns in the mainstream media and online access to unselected information predispose patients with moderate confidence in medicine to turn to alternatives methods of treatment. Personal beliefs and local culture are known to influence treatment adherence [22, 23], including the choice to use alternative medicines. Our patients have at least tried alternative medicines at one point, but that did not make them renounce to traditional medicine.

Some factors like gender, education and the number of prescribed medications are only inconsistently tied to non-adherence; we did not evaluate them in our study [15].

Saturation was rapidly achieved, probably because our study is monocentric and convenience sampling was used, which is one of the limits of the study. In a hospital setting, the patients are reluctant to open, and truthfulness is limited. One other important limit was that the observance assessment was obtained by direct questioning and doctor questioning, with limits of the congruence with the reality.

## Conclusions

In this Romanian population of cirrhotic patients, confidence in medicine is a modifiable extrinsic factor that seems to impact beta blocker prophylaxis adherence negatively. It seems to be counterbalanced by the overall negative perception of cirrhosis, regardless of the real awareness of the disease. Is the answer to the impasse of low observance a more personalized approach to medicine with fully shared decision making or should we simply frighten our patients into submission?
